# Gene Profile of Myeloid-Derived Suppressive Cells from the Bone Marrow of Lysosomal Acid Lipase Knock-Out Mice

**DOI:** 10.1371/journal.pone.0030701

**Published:** 2012-02-27

**Authors:** Cong Yan, Xinchun Ding, Nupur Dasgupta, Lingyan Wu, Hong Du

**Affiliations:** 1 The Center for Immunobiology, Indiana University School of Medicine, Indianapolis, Indiana, United States of America; 2 IU Simon Cancer Center, Indiana University School of Medicine, Indianapolis, Indiana, United States of America; 3 Department of Pathology and Laboratory Medicine, Indiana University School of Medicine, Indianapolis, Indiana, United States of America; 4 Division of Human Genetics, Cincinnati Children's Hospital Medical Center, Cincinnati, Ohio, United States of America; National Cancer Institute, United States of America

## Abstract

**Background:**

Lysosomal acid lipase (LAL) controls development and homeostasis of myeloid lineage cells. Loss of the lysosomal acid lipase (LAL) function leads to expansion of myeloid-derived suppressive cells (MDSCs) that cause myeloproliferative neoplasm.

**Methodology/Principal Findings:**

Affymetrix GeneChip microarray analysis identified detailed intrinsic defects in Ly6G^+^ myeloid lineage cells of LAL knock-out (*lal−/−*) mice. Ingenuity Pathway Analysis revealed activation of the mammalian target of rapamycin (mTOR) signaling, which functions as a nutrient/energy/redox sensor, and controls cell growth, cell cycle entry, cell survival, and cell motility. Loss of the LAL function led to major alteration of large GTPase and small GTPase signal transduction pathways. *lal−/−* Ly6G^+^ myeloid cells in the bone marrow showed substantial increase of cell proliferation in association with up-regulation of cyclin and cyclin-dependent kinase (cdk) genes. The epigenetic microenvironment was significantly changed due to the increased expression of multiple histone cluster genes, centromere protein genes and chromosome modification genes. Gene expression of bioenergetic pathways, including glycolysis, aerobic glycolysis, mitochondrial oxidative phosphorylation, and respiratory chain proteins, was also increased, while the mitochondrial function was impaired in *lal−/−* Ly6G^+^ myeloid cells. The concentration of reactive oxygen species (ROS) was significantly increased accompanied by up-regulation of nitric oxide/ROS production genes in these cells.

**Conclusions/Significance:**

This comprehensive gene profile study for the first time identifies and defines important gene pathways involved in the myeloid lineage cells towards MDSCs using *lal−/−* mouse model.

## Introduction

Myeloid-derived suppressive cells (MDSCs) are heterogeneous populations that express CD11b and Gr-1 antigens. MDSCs actively participate in inflammation-induced pathogenic processes in various diseases (i.e. cancer) by suppressing T lymphocytes [1], [2], [3]. We previously reported that the neutral lipid metabolic pathway controlled by lysosomal acid lipase (LAL) plays a critical role in development and homeostasis of MDSCs [4], [5]. LAL hydrolyzes cholesteryl esters and triglycerides in the lysosome of cells to generate free cholesterol and free fatty acids. Ablating LAL (*lal−/−*) in mice led to aberrant expansion of MDSCs (>40% in the blood, and >70% in the bone marrow) that arise from dysregulated production of myeloid progenitor cells in the bone marrow [4]. Ly6G^+^ MDSCs in *lal−/−* mice show strong immunosuppression on T cells, which contributes to impaired T cell proliferation and function *in vivo* [4], [5]. As a consequence of myeloproliferative neoplasm, severe pathogenic phenotypes in multiple organs are observed in *lal−/−* mice, including the adult lung, liver, spleen, thymus, adrenal glands, and small intestine, which are all associated with MDSCs infiltration [4], [6], [7], [8], [9], [10]. Over-expression of LAL downstream genes in myeloid lineage cells driven by the 7.2 kb c-fms promoter/intron 2 induces chronic inflammation, immunosuppression and tumorigenesis *in vivo* [11], [12], [13].

Given the important role in inflammation and tissue pathogenesis, it is essential to elucidate the intrinsic molecular mechanisms governing MDSCs development and homeostasis in *lal−/−* mice. At the moment, up-regulated genes related to amino acid metabolism (i.e. L-arginine) and production of reactive oxygen (ROS)/nitrogen species are well studied and serve as parameters to define MDSCs [14], [15]. In this report, we aim at identifying a comprehensive gene profile to define pathways that are involved in MDSCs development in *lal−/−* mice by GeneChip microarray analysis. The results showed that the mammalian target of rapamycin (mTOR) signaling, which functions as a nutrient/energy/redox sensor, and controls cell growth, cell cycle entry, cell survival, and cell motility, is activated in bone marrow MDSCs during LAL deficiency.

## Materials and Methods

### Ethics Statement and Animal Care

All scientific protocols involving the use of animals have been approved by the Institutional Animal Care and Use Committee of Indiana University School of Medicine and followed guidelines established by the Panel on Euthanasia of the American Veterinary Medical Association. Protocols involving the use of recombinant DNA or biohazardous materials have been reviewed by the Biosafety Committee of Indiana University School of Medicine and followed guidelines established by the National Institutes of Health. Animals were housed under Institutional Animal Care and Use Committee-approved conditions in a secured animal facility at Indiana University School of Medicine.

### S6 and E4-BP analysis

Fluorescence activated cell sorting (FACS) analysis was performed on single cells from the bone marrow of 5-month-old *lal+/+* and *lal−/−* mice. Bone marrow cells were prepared as previously described [11]. Approximately 1 to 2×10^6^ cells from various organs in FACS buffer were blocked with FcR blocking antibodies (BD Pharmingen, San Diego, CA) followed by incubation with APC anti-mouse CD11b and PE rat anti-mouse Ly6G (1A8, BD Bioscience). Cells were fixed and permeabilized using BD Cytofix/Cytoperm™ Fixation/permeabilization Kit according to the manufacture's instruction, followed by labeling with anti-pS6 (ser235/236) and anti-p4E-BP (Thr37/46) antibodies (1∶50 dilution, Cell Signaling Technology, Beverly, MA) at 4°C overnight. Cells were analyzed on a LSRII machine (BD Biosciences, San Jose, CA). Data were analyzed using the BD FACStation™ Software (BD Biosciences). Quadrants were assigned using isotype control mAb.

### MDSCs RNA isolation

Single cells from bone marrow of 5-month-old *lal+/+* and *lal−/−* mice (n = 5) were stained with anti-Ly6G^+^ antibody, followed by positive magnetic selection using anti-biotin micro-beads following the manufacture's instructions (Miltenyi Biotec, Auburn, CA). The purity of the Ly6G^+^ MDSC population was typically higher than 90%. Total RNAs from isolated Ly6G^+^ MDSCs were purified using the Qiagen total RNA purification kit (Qiagen, Valencia, CA). RNA concentrations were measured with Spectra Max 190 (Molecular Devices, Sunnyvale, CA).

### Affymetrix GeneChip microarray analyses

The quality of the total RNA was checked by the Agilent Bioanalyzer 2100 (Hewelett Packard) using the RNA 6000 Pico Assay. To create biotin labeled cDNA products, 20 nanograms of total RNA was used. Double stranded target cDNA was synthesized using a random hexamer with a T7 promoter. Target sense transcript cRNA was generated from the double-stranded cDNA template using the Whole Transcript cDNA Synthesis and Amplification Kit. cDNA was regenerated using a reverse transcription reaction randomly primed with a mix containing dUTP. After hydrolysis of the cRNA with RNase H, the sense strand of cDNA was purified using the Affymetrix sample cleanup module, fragmented by incubation with UDG (uracil DNA glycosylase) and APE 1 (apurinic/apyrimidicendonuclease 1), and terminally biotin-labeled with TdT (terminal deoxynucleotidyl transferase) using the WT Terminal Labeling Kit. Biotinylated sense strand fragments were hybridized to Affymetrix Mouse Gene 1.0 ST GeneChips using the Hybridization Control and Hybridization Wash and Stain kits at 45°C for 18 hrs. The stained array was scanned using an Affymetrix GeneChip Scanner 3000 7G to generate the CEL files. Primary quality control was performed using the Affymetrix Expression Console. The chip data were imported with Partek Genomics Suite 6.5 (Partek, Inc., St Louis, MO), normalized and summarized using the RMA (Robust Multiarray Average) algorithm. The relative log expression was examined to ensure that the data were properly corrected by normalization and that there were no outliers. Mv A plots were generated to examine the reproducibility of the replicates. To identify expression changes between genotypes, a two-way ANOVA was performed by using the methods of moments [16] to partition the effect of tissue and genotype. Genes differentially expressed in *lal−/−* mice vs *lal+/+* mice were identified at a false positive rate (FDR) of 0.05 and fold change ≥2. The unprocessed microarray data is available at Gene Expression Ominibus Database (GEO) at NCBI (accession number GSE 29401).

### Pathway and functional classification

Significantly affected or differentially expressed genes were subjected to an intensive search to identify biological functions. Functional pathway gene ontology and network analysis were executed using Ingenuity Pathway Analysis (IPA) (Ingenuity Systems, Mountain View, CA), Partek (Partek Inc., St. Louis, MO), public information and literature references. The enriched functional categories were determined by Fisher Exact Test using the corresponding murine genome as a reference dataset. The significance was set at *p*-value<0.05. The differentially expressed genes were grouped into various categories. To cluster gene expression profiles, Hierarchical cluster analysis of the significantly expressed genes was performed using Partek genomic suite 6.6 (St. Louis, MO) which showed the correlated groups of genes and their expression patterns across all points.

### FACS for JC-1 analysis

Mitochondrial membrane potential was measured according to MitoProbe JC-1 Assay Kit (Molecular Probe, Eugene, OR). Bone marrow single cells from *lal+/+* and *lal−/−* mice were suspended in pre-warmed PBS. Cells were then stained with anti-CD11b, anti-Ly6G antibody and 10 µl of 200 µM JC-1 dye. After incubation at 37°C, 5% CO_2_ for 30 minutes, cells were suspended by gently flicking the tubes with the warmed PBS and washed twice. JC-1 was analyzed by flow cytometry in gated CD11b^+^Ly6G^+^ cells.

### ROS detection

Ly6G^+^ cells were isolated from the bone marrow of *lal+/+* and *lal−/−* mice with ly6G^+^ antibody-coated magnetic beads and MACS-LS columns according to the manufacturer's instructions (Miltenyi Biotec, Auburn, CA). Purified cells were suspended in pre-warmed PBS. Oxidation-sensitive dye 2′-7′-dichlorodihydrofluorescein diacetate (DCFDA, C6827, Invitrogen, Eugene, OR), was used to measure ROS production by MDSC. Cells were incubated at 37°C in pre-warmed PBS in the presence of 2.5 µM DCFDA for 20 min. Cells were then labeled with APC-conjugated anti-Ly6G^+^ and PE-conjugated anti-CD11b Abs on ice. Analysis was then conducted by flow cytometry.

### Cell Cycle

Bone marrow cells from *lal+/+* and *lal−/−* mice were stained with surface markers anti-CD11b and ly6G antibodies (1∶200 dilution, BD Bioscience) for 15 min at 4°C. After washed with PBS, cells were fixed in 1 ml 70% methanol for 1 hour at room temperature. After cells were washed with PBS, 100 µl of 10 mg/ml RNAase (Rase LS005649, Worthington Biochemicals, Lakewood, NJ) and 1 ml of 50 µg/ml propidium iodide (PI, P4170, Sigma, Saint Louis, MI) were added and incubated for 30 min at 37°C. Cells were washed with PBS. PI-labeled DNA concentrations were analyzed in gated CD11b^+^Ly6G^+^ cells by FACS Calibur APC machine. The data were analyzed with ModFit LT^Tm^ DNA analysis software (VMFLTMAC3, Verity Software House, Topsham, ME).

### ATP assay

Bone marrow cells (1×10^6^) were rinsed with PBS and lysed with ATP-releasing buffer containing 100 mM potassium phosphate buffer (pH 7.8), 2 mM EDTA, 1 mM dithiothreitol, and 1% Triton X-100. The ATP concentration of cell lysate was measured using ATP kit (Invitrogen, Carlsbad, CA) according to the manufacture's instruction.

## Results

### Affymetrix GeneChip microarray and Ingenuity pathway analyses of Ly6G^+^ cells from the lal−/− bone marrow

**Figure 1:**
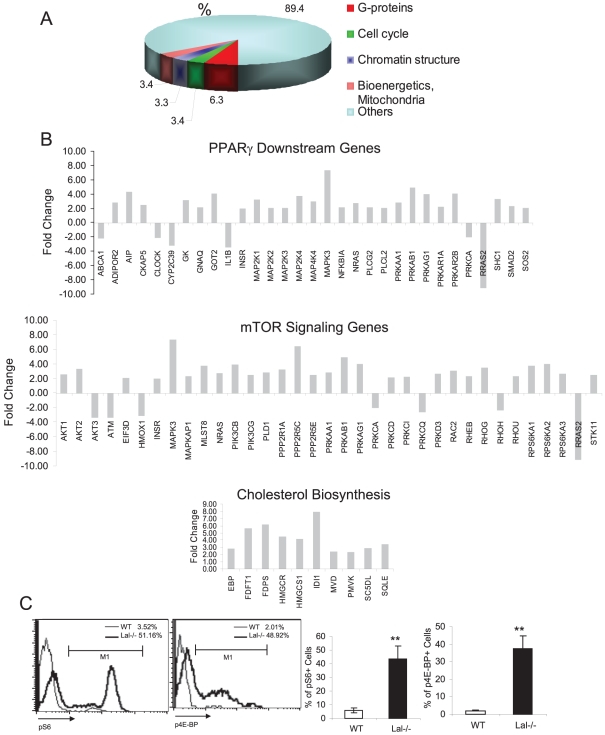
Affymetrix GeneChip microarray and Ingenuity Pathway analyses of lal−/− MDSCs. **A**). Affymetrix GeneChip microarray analysis of Ly6G^+^ MDSCs from the bone marrow of *lal+/+* mice and lal−/− mice. Numbers represent percentages of changed genes in each category vs total changed genes between *lal−/−* mice and *lal+/+* mice; **B**). Differential gene expression of Ly6G^+^ MDSCs from the bone marrow of *lal+/+* mice and *lal−/−* mice was analyzed by Ingenuity Pathway Analysis. Changed genes in the PPARγ pathway, cholesterol biosynthesis and mTOR signaling pathway are presented; **C**) Flow cytometry of mTOR downstream effectors S6 and 4E-BP1 in Ly6G^+^ MDSCs from the bone marrow of *lal+/+* mice and *lal−/−* mice. n = 4.

In order to identify intrinsic defects of MDSCs in *lal−/−* mice, total RNAs were isolated from Ly6G^+^ myeloid cells of the age-matched wild type and *lal−/−* bone marrow. Purified total RNAs were subjective to the Affymetrix GeneChip microarray study. At a false positive rate (FDR) of 0.05 and fold change ≥2, there were 3086 changed genes in *lal−/−* bone marrow MDSCs. Major changes of *lal−/−*bone marrow MDSCs were observed in G-protein signaling, cell cycle, chromatin modification and bioenergetics (Figure 1A). Gene expression of both Ly6G (9.1 fold) and Ly6C (7.1 fold) was increased in *lal−/−* bone marrow MDSCs. Ingenuity Pathway Analysis revealed alteration of several important pathways in *lal−/−* bone marrow MDSCs (Figure 1B). Compared with normal wild-type cells, expression of genes that are positively or negatively regulated by LAL downstream effector PPARγ was altered. Due to lack of cholesterol generation through the LAL pathway, genes involved in cholesterol biosynthesis were increased in *lal−/−* bone marrow MDSCs to compensate the loss. Importantly, genes involved in the mammalian target of rapamycin (mTOR) singaling pathway were altered in *lal−/−* bone marrow MDSCs. The mTOR is a serine/threonine protein kinase that regulates cell growth, cell proliferation, cell motility, cell survival, protein synthesis, and transcription in response to growth factors, and mitogens. mTOR also senses cellular nutrient and energy levels and redox status [17]. Compared with wild type mice, mTOR downstream effectors S6 (S6) and 4E-BP1 were highly phosphorylated in *lal−/−* bone marrow MDSCs (Figure 1C), a strong indication of mTOR activation.

### ROS production in lal−/− bone marrow MDSCs

**Figure 2:**
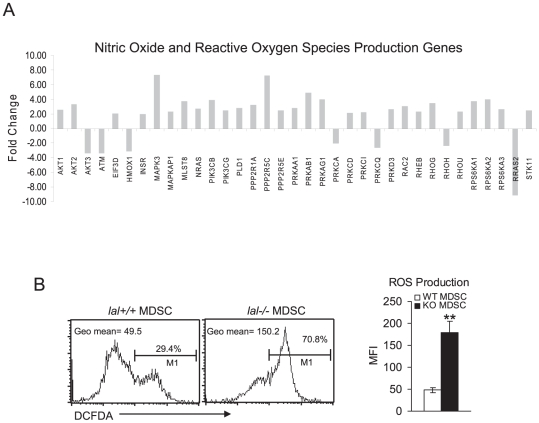
ROS Pathway Analysis of lal−/− bone marrow MDSCs. **A**). Differential gene expression of CD11b^+^Ly6G^+^ MDSCs from the bone marrow of *lal+/+* mice and *lal−/−* mice was analyzed by Ingenuity Pathway Analysis. Changed genes in the nitric oxide and reactive oxygen species production genes are presented; **B**). ROS production in CD11b^+^Ly6G^+^ MDSCs from the bone marrow of *lal+/+* mice and *lal−/−* mice. The ROS signal was statistically analyzed by mean fluorescent intensity (MFI). Results are means of 4 independent FACS experiments. n = 4, **, p<0.01.

Since mTOR controls the redox status, and nitric oxide and reactive oxygen species production are known to play important roles in MDSCs, genes involved in nitric oxide and ROS production were investigated in *lal−/−* bone marrow MDSCs. As demonstrated in Figure 2A, expression of these genes was significantly altered. As a result, the concentration of ROS was increased 3 times in the Ly6G population of *lal−/−* bone marrow MDSCs compared with those in wild type MDSCs (Figure 2B). Reduced glutathione (GSH) protects the cell by destroying hydrogen peroxide and hydroxyl free radicals. Both glutathione peroxidase (3.0 fold) and glutathione reductase genes (20.1 fold) showed increased expression in *lal−/−* bone marrow MDSCs as well (Table S2). The gene expression level of glucose 6-phosphate dehydrogenase that is required for regeneration of GSH from its oxidized form (GSSG) was increased in *lal−/−* bone marrow MDSCs as well (Table S2).

### Gene expression levels of G-protein family in lal−/− MDSCs

Guanosine nucleotide-binding proteins (G proteins) are involved in many aspects of cellular functions. They play key roles in signal transduction and regulation of gene expression in almost all cell types including immune cells [18]. This group of proteins can be separated into two classes. The first class is the heterotrimeric G protein superfamily, consisting of α-, β-, and γ-subunit, with the α-subunit binding the guanosine nucleotides. Many members in this large G-protein superfamily, including receptors, effectors, heterotrimeric Gαβγ subunits, and the regulators of G-protein signaling proteins were altered in gene expression in *lal−/−* bone marrow MDSCs (Table 1, 2). The second class is the monomeric low-molecular-weight Ras GTPase superfamily, which contains more than 100 proteins, and cycles between an inactive guanosine diphosphate (GDP) and an active GTP-bound form [19]. As demonstrated in Table 3, 4, 5, gene expression of many members of the small GTPase superfamily and downstream effectors were altered in *lal−/−* bone marrow MDSCs. It is known that G-protein family has a tight relationship with the mTOR activity [20].

**Table 1:** Increases of large G-protein superfamily in *lal−/−* bone marrow MDSCs.

Genes	Symbol	Fold
G protein-coupled receptor 137B, pseudogene	Gpr137b-ps	11.6
regulator of G-protein signaling 18	Rgs18	9.5
G protein-coupled receptor 84	Gpr84	9.1
G protein-coupled receptor 141	Gpr141	6.3
G protein-coupled receptor 97	Gpr97	5.9
guanylate cyclase activator 1a (retina)	Guca1a	3.6
regulator of G-protein signaling 19	Rgs19	3.4
G protein-coupled receptor 108	Gpr108	3.4
GTP-binding protein 8 (putative)	Gtpbp8	3.4
IQ motif containing GTPase activating protein 2	Iqgap2	3.3
CAP, adenylate cyclase-associated protein 1 (yeast)	Cap1	3.0
G protein-coupled receptor 107	Gpr107	2.7
guanine nucleotide binding protein (G protein), gamma 12	Gng12	2.7
G protein-coupled receptor kinase 6	Grk6	2.7
Guanosine diphosphate (GDP) dissociation inhibitor 1	Gdi1	2.7
guanine nucleotide binding protein (G protein), beta 1	Gnβ1	2.6
GTP binding protein 1	Gtpbp1	2.6
guanine nucleotide binding protein (G protein), alpha inhibitor 2	Gnαi2	2.5
guanine nucleotide binding protein (G protein), beta 5	Gnβ5	2.4
ArfGAP with GTPase domain, ankyrin repeat and PH domain 1	Agap1	2.3
G-protein signalling modulator 2 (AGS3-like, C. elegans)	Gpsm2	2.2
developmentally regulated GTP binding protein 1	Drg1	2.2
SLIT-ROBO Rho GTPase activating protein 2	Srgap2	2.2
RAP1, GTP-GDP dissociation stimulator 1	Rap1gds1	2.2
G-protein signalling modulator 3 (AGS3-like, C. elegans)	Gpsm3	2.1
guanine nucleotide binding protein, alpha q polypeptide	Gnαq	2.1
guanine nucleotide binding protein (G protein), beta 2	Gnβ2	2.0
guanine nucleotide binding protein (G protein), gamma 5	Gnγ5	2.0

**Table 2:** Decreases of large G-protein superfamily in *lal−/−* bone marrow MDSCs.

Genes	Symbol	Fold
guanine nucleotide binding protein (G protein), gamma tran	Gnγt2	−2.0
guanylate binding protein 4	Gbp4	−2.2
regulator of G-protein signalling 10	Rgs10	−2.2
GTP binding protein (gene overexpressed in skeletal muscle)	Gem	−2.4
SLIT-ROBO Rho GTPase activating protein 3	Srgap3	−2.5
guanylate binding protein 3	Gbp3	−2.5
GTPase, IMAP family member 8	Gimap8	−2.8
purinergic receptor P2Y, G-protein coupled, 14	P2ry14	−3.0
GTPase, IMAP family member 1	Gimap1	−4.0
guanine nucleotide binding protein-like 3 (nucleolar)	Gnl3	−4.1
Rap guanine nucleotide exchange factor (GEF) 4	Rapgef4	−4.6
G protein-coupled receptor 18	Gpr18	−5.5
GTPase, very large interferon inducible 1 pseudogene	Gm4759	−5.8
guanylate binding protein 1	Gbp1	−5.9
G protein-coupled receptor 183	Gpr183	−7.5
G protein-coupled receptor 174	Gpr174	−7.6
GTPase, IMAP family member 4	Gimap4	−9.0
G protein-coupled receptor 171	Gpr171	−9.2
GTPase, IMAP family member 6	Gimap6	−14.0

**Table 3:** Increases of monomeric low-molecular-weight Ras GTPase superfamily in *lal−/−* bone marrow MDSCs.

Genes	Symbol	Fold
RAB3D, member RAS oncogene family	Rab3d	7.5
mitogen-activated protein kinase 3	Mapk3	7.3
Shc SH2-domain binding protein 1	Shcbp1	6.8
Rho GTPase activating protein 19	Arhgap19	6.4
mitogen-activated protein kinase-activated protein kinase 3	Mapkapk3	6.0
multiple EGF-like-domains 9	Megf9	5.9
RAB1B, member RAS oncogene family	Rab1b	5.4
mitogen-activated protein kinase 13	Mapk13	5.1
RAB37, member of RAS oncogene family	Rab37	5.0
p21 protein (Cdc42/Rac)-activated kinase 1	Pak1	4.9
Ras and Rab interactor 2	Rin2	4.9
RAB11B, member RAS oncogene family	Rab11b	4.7
RAB5B, member RAS oncogene family	Rab5b	4.4
MAP kinase-activated protein kinase 2	Mapkapk2	4.3
RAB18, member RAS oncogene family	Rab18	4.2
RAS related protein 2a	Rap2a	4.1
RAB28, member RAS oncogene family	Rab28	4.0
Rho guanine nucleotide exchange factor (GEF) 12	Arhgef12	4.0
RAB interacting factor	Rabif	3.8
mitogen-activated protein kinase kinase 4	Map2k4	3.8
thymoma viral proto-oncogene 1 interacting protein	Aktip	3.8
neuroblastoma ras oncogene	Nras	2.8
RAB31, member RAS oncogene family	Rab31	3.6
ras homolog gene family, member G	Rhog	3.5
CDC42 effector protein (Rho GTPase binding) 3	Cdc42ep3	3.5
Rab interacting lysosomal protein-like 2	Rilpl2	3.4
RAB27A, member RAS oncogene family	Rab27a	3.4
RAB24, member RAS oncogene family	Rab24	3.3
thymoma viral proto-oncogene 2	Akt2	3.3
mitogen-activated protein kinase kinase 1	Map2k1	3.2
JNK1/MAPK8-associated membrane protein	Jkamp	3.2
RAB18, member RAS oncogene family	Rab18	3.1
RAS-related C3 botulinum substrate 2	Rac2	3.1
mitogen-activated protein kinase kinase kinase kinase 4	Map4k4	3.0
mitogen-activated protein kinase kinase kinase 9	Map3k9	3.0
Rho GTPase activating protein 1	Arhgap1	3.0
RAB5C, member RAS oncogene family	Rab5c	2.9
RAB23, member RAS oncogene family	Rab23	2.8
Ras suppressor protein 1	Rsu1	2.8
Ras-like without CAAX 1	Rit1	2.8
Rac GTPase-activating protein 1	Racgap1	2.8

**Table 4:** Increases of additional monomeric low-molecular-weight Ras GTPase superfamily in *lal−/−* bone marrow MDSCs.

Genes	Symbol	Fold
mitogen-activated protein kinase 7	Mapk7	2.7
RAB22A, member RAS oncogene family	Rab22a	2.6
thymoma viral proto-oncogene 1	Akt1	2.6
MAPK scaffold protein 1	Mapksp1	2.5
Rho GTPase activating protein 30	Arhgap30	2.5
RAB11a, member RAS oncogene family	Rab11a	2.4
MAPK scaffold protein 1	Mapksp1	2.4
mitogen-activated protein kinase 6	Mapk6	2.4
Rac/Cdc42 guanine nucleotide exchange factor (GEF) 6	Arhgef6	2.4
RAB7, member RAS oncogene family	Rab7	2.3
RAB4B, member RAS oncogene family	Rab4b	2.3
ras homolog gene family, member U	Rhou	2.3
RAB6, member RAS oncogene family	Rab6	2.3
RAB32, member RAS oncogene family	Rab32	2.3
mitogen-activated protein kinase associated protein 1	Mapkap1	2.3
MAF1 homolog (S. cerevisiae)	Maf1	2.3
Ras homolog enriched in brain	Rheb	2.3
Ras association (RalGDS/AF-6) domain family member 5	Rassf5	2.3
ArfGAP with GTPase domain, ankyrin repeat and PH domain 1	Agap1	2.3
RAS guanyl releasing protein 4	Rasgrp4	2.3
RAB guanine nucleotide exchange factor (GEF) 1	Rabgef1	2.3
Rho family GTPase 1	Rnd1	2.2
Ras association (RalGDS/AF-6) domain family member 3	Rassf3	2.2
MAP kinase-activated protein kinase 5	Mapkapk5	2.2
mitogen-activated protein kinase kinase kinase kinase 2	Map4k2	2.2
Ras and Rab interactor-like	Rinl	2.2
RAB35, member RAS oncogene family	Rab35	2.2
Srgap2//SLIT-ROBO Rho GTPase activating protein 2	Srgap2	2.2
Rho GTPase activating protein 4	Arhgap4	2.2
Rho GTPase activating protein 11A	Arhgap11a	2.2
RAP1, GTP-GDP dissociation stimulator 1	Rap1gds1	2.2
mitogen-activated protein kinase kinase kinase 3	Map3k3	2.1
mitogen-activated protein kinase kinase 3	Map2k3	2.1
mitogen-activated protein kinase kinase 2	Map2k2	2.1
Rab acceptor 1 (prenylated)	Rabac1	2.1
Rho GTPase activating protein 15	Arhgap15	2.1
farnesyltransferase, CAAX box, alpha	Fntα	2.0
RAP2C, member of RAS oncogene family	Rap2c	2.0
RAB43, member RAS oncogene family	Rab43	2.0
RAB3 GTPase activating protein subunit 2	Rab3gap2	2.0

**Table 5:** Decreases of monomeric low-molecular-weight Ras GTPase superfamily in *lal−/−* bone marrow MDSCs.

Genes	Symbol	Fold
ras homolog gene family, member H	Rhoh	−2.4
SLIT-ROBO Rho GTPase activating protein 3	Srgap3	−2.5
ras homolog gene family, member Q	Rhoq	−2.7
Rho GTPase activating protein 12	Arhgap12	−3.4
thymoma viral proto-oncogene 3	Akt3	−3.4
RasGEF domain family, member 1B	Rasgef1b	−3.5
Rap guanine nucleotide exchange factor (GEF) 4	Rapgef4	−4.6
RAS guanyl releasing protein 1	Rasgrp1	−4.7
RAB30, member RAS oncogene family	Rab30	−5.2
RAS, guanyl releasing protein 3	Rasgrp3	−10.2

### Gene expression levels of cell cycle protein family in lal−/− MDSCs

**Figure 3:**
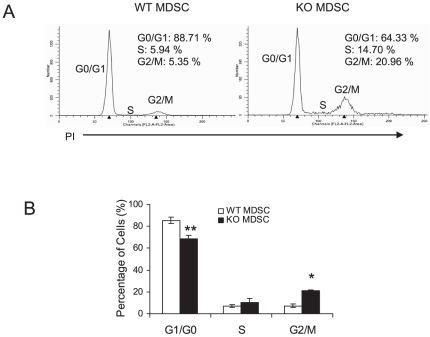
Cell cycle analysis of lal−/− bone marrow MDSCs. **A**). Cell cycle analysis of CD11b^+^ly6G^+^ MDSCs from the bone marrow of *lal+/+* mice and *lal−/−* mice; **B**). The G1/G0 phase, S phase and G2/M phase of CD11b^+^Ly6G^+^ MDSCs from the bone marrow of *lal+/+* mice and *lal−/−* mice were statistically analyzed. Results are means of 4 independent FACS experiments. n = 4, **, p<0.01, *, p<0.5.

The cell cycle consists of four distinct phases: G_1_ phase, S phase (synthesis), G_2_ phase and M phase (mitosis). The G_1_ checkpoint control mechanism ensures that everything is ready for DNA synthesis. DNA replication occurs during the S phase. During the interphase (cell grows), nutrients are accumulated and DNA is duplicated in cells. The G_2_ checkpoint control mechanism ensures that everything is ready to enter the M phase and divide. During the mitosis (M) phase, the cell splits itself into two distinct daughter cells. The cell cycle is precisely controlled by cyclin-dependent kinases (CDKs). CDK activity requires binding of regulatory subunit cyclins [21]. When activated by a bound cyclin, CDKs perform phosphorylation that activates or inactivates target proteins to orchestrate coordinated entry into the next phase of the cell cycle. Gene expression of many Cdk and cyclin family members of the cell cycle were altered in *lal−/−* bone marrow MDSCs (Table 6). In *lal−/−* bone marrow MDSCs, gene expression of all four critical cyclins (A, B, D, E-type) as well as Cdk1, Cdk2, Cdk5, Cdk9 were up-regulated compared with wild type MDSCs (Table 6). When the cell cycle was studied, 4 times more *lal−/−* MDSCs (20.96%) were accumulated in the G2/M phases than wild type MDSCs (5.35%) (Figure 3A, B), suggesting that *lal−/−* bone marrow MDSCs are highly proliferative. This is consistent with our previous BrdU proliferation study [4].

**Table 6:** Up-regulation of cyclin proteins in *lal−/−* bone marrow MDSCs.

Genes	Symbol	Fold
cyclin-dependent kinase inhibitor 3	Cdkn3	8.4
cyclin B1	Ccnb1	7.2
cell division cycle 2 homolog A (S. pombe)	Cdc2a	6.6
cyclin B2	Ccnb2	5.6
cell division cycle associated 8	Cdca8	5.4
cyclin A2 (S to G2 transition, KO embryonic lethality)	Ccna2	5.1
meiotic nuclear divisions 1 homolog (S. cerevisiae)	Mnd1	4.5
cell growth regulator with ring finger domain 1	Cgrrf1	4.4
cyclin-dependent kinase inhibitor 2D	Cdkn2d	4.2
cyclin-dependent kinase 2 (G1 to S transition)	Cdk2	4.0
cyclin-dependent kinase 5	Cdk5	4.0
CDC14 cell division cycle 14 homolog A (S. cerevisiae)	Cdc14a	3.8
cell division cycle associated 3	Cdca3	3.5
cell division cycle 45 homolog (S. cerevisiae)-like	Cdc45l	3.4
cyclin-dependent kinase inhibitor 2C (p18, inhibits CDK4)	Cdkn2c	3.4
cyclin C	Ccnc	3.3
cell division cycle 25 homolog C (S. pombe)	Cdc25c	3.3
cell division cycle associated 7	Cdca7	3.2
cell division cycle 25 homolog A (S. pombe).	Cdc25a	2.9
cell division cycle 20 homolog (S. cerevisiae)	Cdc20	2.9
cell division cycle 26	Cdc26	2.7
cyclin D3	Ccnd3	2.6
cell division cycle associated 2	Cdca2	2.6
CDC42 small effector 1	Cdc42se1	2.5
cyclin-dependent kinase 9 (CDC2-related kinase)	Cdk9	2.5
cell division cycle 34 homolog (S. cerevisiae)	Cdc34	2.4
cell division cycle 2-like 1	Cdc2l1	2.4
cell division cycle 123 homolog (S. cerevisiae)	Cdc123	2.3
cyclin-dependent kinase-like 2 (CDC2-related kinase)	Cdkl2	2.3
cyclin E2 (G1 to S transition)	Ccne2	2.3
cyclin-dependent kinase-like 2 (CDC2-related kinase)	Cdkl2	2.3
cell division cycle 2-like 6 (CDK8-like)	Cdc2l6	2.3
CDC42 small effector 2	Cdc42se2	2.2
protein interacting with cyclin A1	Proca1	2.3
cyclin G2	Ccng2	2.2
cell division cycle 37 homolog (S. cerevisiae)	Cdc37	2.1
cyclin I	Ccni	2.1
cyclin B1 interacting protein 1	Ccnb1ip1	−2.2
cyclin D2	Ccnd2	−2.4
growth arrest specific 5	Gas5	−3.3

The G_1_ cyclin-CDK complexes also promote the degradation of molecules (i.e. cyclins after using) that function as S phase inhibitors by targeting them for ubiquitination [21]. Once a protein has been ubiquitinated, it is targeted for proteolytic degradation by the proteasome. In *lal−/−* bone marrow MDSCs, gene expression of enzymes/protein factors involved in ubiquitination (Table 7) and proteasome subunits (Table 8) showed up-regulation, indicating an enhanced protein recycling activity.

**Table 7:** Gene expression changes of ubiquitination related proteins in *lal−/−* bone marrow MDSCs.

Genes	Symbol	Fold
ubiquitin specific peptidase 46	Usp46	5.4
OTU domain, ubiquitin aldehyde binding 1	Otub1	4.7
AN1, ubiquitin-like, homolog (Xenopus laevis)	Anubl1	4.0
ubiquitin-conjugating enzyme E2G 2	Ube2g2	3.9
ubiquitin specific peptidase 39	Usp39	3.7
ubiquitin-like 4	Ubl4	3.5
ubiquitin-conjugating enzyme E2, J2 homolog (yeast)	Ube2j2	3.0
ubiquitin-conjugating enzyme E2A, RAD6 homolog (S. cerevis	Ube2a	2.9
ubiquitin-conjugating enzyme E2S	Ube2s	2.9
ubiquitin fusion degradation 1 like	Ufd1l	2.9
ubiquitin-like domain containing CTD phosphatase 1	Ublcp1	2.9
ubiquitin-conjugating enzyme E2N	Ube2n	2.8
ubiquitin-fold modifier conjugating enzyme 1	Ufc1	2.7
ubiquitin-conjugating enzyme E2H	Ube2h	2.6
ubiquitin-associated protein 1	Ubap1	2.6
ubiquitin-conjugating enzyme E2C	Ube2c	2.5
ubiquitin specific peptidase 1	Usp1	2.4
WW domain containing E3 ubiquitin protein ligase 2	Wwp2	2.4
ubiquitin-fold modifier 1	Ufm1	2.4
similar to ubiquitin-conjugating enzyme E2N	LOC635086	2.3
ubiquitin-like modifier activating enzyme 3	Uba3	2.3
ubiquitin-like modifier activating enzyme 2	Uba2	2.2
ubiquitin interaction motif containing 1	Uimc1	2.2
ubiquitin specific peptidase 5 (isopeptidase T)	Usp5	2.2
ubiquitin-conjugating enzyme E2 variant 1	Ube2v1	2.2
ubiquitin-like 7 (bone marrow stromal cell-derived)	Ubl7	2.2
ubiquitin-like 3	Ubl3	2.1
ubiquitin-like modifier activating enzyme 1	Uba1	2.1
ubiquitin-conjugating enzyme E2, J1	Ube2j1	2.1
ubiquitin specific peptidase 22	Usp22	2.1
HECT domain and ankyrin repeat containing, E3 ubiquitin pr	Hace1	2.0
transmembrane and ubiquitin-like domain containing 2	Tmub2	2.0
ubiquitin protein ligase E3 component n-recognin 2	Ubr2	2.0
ubiquitin-conjugating enzyme E2M (UBC12 homolog, yeast)	Ube2m	2.0
ubiquitin specific peptidase 31	Usp31	−2.2
similar to ubiquitin-conjugating enzyme E2 va	LOC635992	−2.6
ubiquitin D	Ubd	−4.6

**Table 8:** Gene expression changes of proteasome protein subunits in *lal−/−* bone marrow MDSCs.

Genes	Symbol	Fold
proteasome (prosome, macropain) subunit, beta type 3	Psmb3	8.5
proteasome (prosome, macropain) 26S subunit, non-ATPase,	Psmd13	4.4
proteasome (prosome, macropain) 26S subunit, non-ATPase,	Psmd10	3.6
proteasome (prosome, macropain) 26S subunit, non-ATPase, 8	Psmd8	3.3
proteasome (prosome, macropain) 26S subunit, non-ATPase, 4	Psmd4	3.1
proteasome (prosome, macropain) subunit, beta type 1	Psmb1	3.0
proteasome (prosome, macropain) 26S subunit, non-ATPase, 5	Psmd5	2.9
proteasome (prosome, macropain) subunit, beta type 6	Psmb6	2.7
proteasome (prosome, macropain) subunit, beta type 2	Psmb2	2.6
proteasome (prosome, macropain) subunit, alpha type 2	Psma2	2.5
proteasome (prosome, macropain) subunit, beta type 5	Psmb5	2.5
proteasome (prosome, macropain) 26S subunit, ATPase, 6	Psmc6	2.5
proteasome (prosome, macropain) subunit, beta type 7	Psmb7	2.4
proteasome (prosome, macropain) subunit, alpha type 1	Psma1	2.4
proteasome (prosome, macropain) 28 subunit, beta	Psme2	2.4
proteasome (prosome, macropain) subunit, alpha type 7	Psma7	2.3
proteasome (prosome, macropain) 26S subunit, ATPase, 4	Psmc4	2.2
proteasome (prosome, macropain) 26S subunit, non-ATPase, 6	Psmd6	2.2
proteasome (prosome, macropain) subunit, beta type 4	Psmb4	2.1
proteasome (prosome, macropain) 26S subunit, non-ATPase, 7	Psmd7	2.1
proteasome (prosome, macropain) 26S subunit, non-ATPase, 2	Psmd2	2.1
proteasome (prosome, macropain) 26S subunit, ATPase 2	Psmc2	2.1
proteasome (prosome, macropain) assembly chaperone 1	Psmg1	2.1
proteasome (prosome, macropain) subunit, alpha type 4	Psma4	2.1

### Gene expression levels of histone cluster protein family and centromere protein family in lal−/− MDSCs

Cell proliferation and cell cycle are also controlled by the chromatin activity. The nucleosomes form the basic repeating units of chromatin in eukaryotes. The composition of the individual nucleosomes is fundamentally similar and consists of an octameric core of four types of histones (H2A, H2B, H3 and H4). The expression of the major histones is tightly regulated during the cell cycle, and the histones are deposited onto DNA in a process that is strictly coupled to DNA replication. However, histone variants are not subject to this stringent regulation, and are expressed throughout the cell cycle. Histone-variant exchange activities contribute to gene expression and other cellular events (i.e. formation of centromeric and telomeric chromatin during cell cycles) [22]. In *lal−/−* bone marrow MDSCs, gene expression of multiple histone variants were increased (Table 9). The function of histone deposition onto DNA is also influenced by interacting with other associated proteins and by posttranslational modification (such as phosphorylation, methylation, and acetylation, polyADP ribosylation and monoubiquitylation) [22]. Alteration of gene expression of these factors was observed in *lal−/−* bone marrow MDSCs (Table 7 and Table S1).

**Table 9:** Up-regulation of histone cluster genes in *lal−/−* bone marrow MDSCs.

Genes	Symbol	Fold
histone cluster 1, H4d	Hist1h4d	11.2
histone cluster 1, H4m	Hist1h4m	9.4
histone cluster 1, H4f	Hist1h4f	9.3
histone cluster 1, H2bc	Hist1h2bc	8.1
histone cluster 1, H4b	Hist1h4b	7.6
histone cluster 2, H4	Hist2h4	7.4
histone cluster 1, H2be	Hist1h2be	6.7
histone cluster 1, H2bb	Hist1h2bb	5.8
histone cluster 1, H2ab	Hist1h2ab	5.2
histone cluster 1, H2an	Hist1h2an	4.3
histone cluster 1, H2bm	Hist1h2bm	4.2
histone cluster 1, H3g	Hist1h3g	4.0
histone cluster 1, H3f	Hist1h3f	3.9
histone cluster 1, H2ao	Hist1h2ao	3.8
H1 histone family, member 0	H1f0	3.7
histone cluster 1, H2af	Hist1h2af	3.7
histone cluster 4, H4	Hist4h4	3.6
H2A histone family, member X	H2afx	3.6
histone cluster 1, H3a	Hist1h3a	3.5
histone cluster 2, H3b	Hist2h3b	3.5
histone cluster 2, H2aa1	Hist2h2aa1	3.5
H2A histone family, member Y2	H2afy2	3.1
histone cluster 2, H2bb	Hist2h2bb	3.1
histone cluster 2, H3c1	Hist2h3c1	2.8
histone cluster 1, H2ak	Hist1h2ak	2.7
H2A histone family, member Y	H2afy	2.7
histone cluster 1, H4c	Hist1h4c	2.6
histone cluster 1, H1b	Hist1h1b	2.6
histone cluster 1, H2bh	Hist1h2bh	2.3
histone cluster 1, H2bg	Hist1h2bg	2.2
H2A histone family, member Z	H2afz	2.1
H3 histone, family 3B	H3f3b	2.0
histone cluster 1, H1a	Hist1h1a	2.0

During the M phase of cell cycle, the centromeric histones (i.e. CENPA) are required for chromosome segregation. They form centromere that is the site of spindle attachment to the chromosomes during mitosis. Gene expression of multiple centromeric and telomeric histones along with other M phase chromosome structural proteins (i.e. kinetochore protein subunits) was increased in *lal−/−* bone marrow MDSCs (Table 10).

**Table 10:** Gene expression changes of centromere protein and chromosome structural genes in *lal−/−* bone marrow MDSCs.

Genes	Symbol	Fold
centromere protein H	Cenph	6.1
centromere protein F	Cenpf	4.2
centromere protein Q	Cenpq	3.8
centromere protein K	Cenpk	3.6
centromere protein J	Cenpj	3.5
centromere protein L	Cenpl	3.4
centromere protein E	Cenpe	3.2
centromere protein P	Cenpp	2.8
centromere protein A	Cenpa	2.7
centromere protein O	Cenpo	2.2
NUF2, NDC80 kinetochore complex component, homolog (S. cerevisia	Nuf2	6.5
SPC25, NDC80 kinetochore complex component, homolog (S. cerevisia	Spc25	6.5
SPC24, NDC80 kinetochore complex component, homolog (S. cerevisia	Spc24	6.1
NDC80 homolog, kinetochore complex component (S. cerevisia	Ndc80	3.6
kinetochore associated 1	Kntc1	2.1
Zwilch, kinetochore associated, homolog (Drosophila)	Zwilch	2.1
Centrosomal protein 55	Cep55	4.9
nucleolar and spindle associated protein 1	Nusap1	3.8
structural maintenance of chromosomes 2	Smc2	3.6
spindle assembly 6 homolog (C. elegans)	Sass6	3.5
chromatin modifying protein 4B	Chmp4b	2.6
protection of telomeres 1A	Pot1a	2.5
chromatin modifying protein 2A	Chmp2a	2.4
inner centromere protein	Incenp	2.3
chromatin modifying protein 5	Chmp5	2.3
regulator of chromosome condensation (RCC1) and BTB (POZ)	Rcbtb2	2.2
chromatin accessibility complex 1	Chrac1	2.2
Centrosome and spindle pole associated protein 1	Cspp1	2.1
chromatin modifying protein 1B	Chmp1b	2.1
chromodomain helicase DNA binding protein 7	Chd7	2.0
regulator of chromosome condensation (RCC1) and BTB (POZ)	Rcbtb1	−2.4

### Gene expression levels of metabolic proteins involved in glycolysis and citric acid cycles in lal−/− MDSCs

Metabolism provides the cell with the energy and resources to support cell growth. Glucose serves as a fuel for ATP generation. Glycolysis occurs in the cytosol. The link between glycolysis and citric acid cycle is the oxidative decarboxylation of pyruvate to form acetyl CoA. In the matrix of mitochondrial, the citric acid cycle is the final common pathway for the oxidation of fuel molecules. It also serves as a source of building blocks for biosyntheses. Many enzymes that are involved in the glycolytic pathway (hexokinase, glucose phosphate isomerase, aldolase, triosephosphate isomerase, glyceraldehyde-3-phosphate dehydrogenase, phosphoglycerate kinase, enolase, pyruvate kinase) and the citric acid cycle (isocitrate dehydrogenase 1, succinate dehydrogenase complex, subunit B, malate dehydrogenase 2) were up-regulated in *lal−/−* bone marrow MDSCs (Table 11). In addition, gene expression of both lactate dehydrogenase A and B was up-regulated in *lal−/−* bone marrow MDSCs (Table 11), a pathway generally used by cancer cells (Warburg effect). Some enzymes in the pentose phosphate pathway (energy conservation for biosynthetic purposes) and glycogen synthesis (storage form of glucose and metabolic energy) were also up-regulated in *lal−/−* bone marrow MDSCs (Table S2).

**Table 11:** Up-regulation of metabolic enzyme genes in glycolysis, citric acid cycle and glycogen syntheses in *lal−/−* bone marrow MDSCs.

Genes of glycolysis	Symbol	Fold
hexokinase 2	Hk2	3.8
hexokinase 1	Hk1	3.2
hexokinase 3	Hk3	2.7
glucose phosphate isomerase 1	Gpi1	5.2
aldolase A, fructose-bisphosphate	Aldoa	2.9
fructose bisphosphatase 1	Fbp1	11.0
triosephosphate isomerase 1	Tpi1	3.3
glyceraldehyde-3-phosphate dehydrogenase	Gapdh	5.0
phosphoglycerate kinase 1	Pgk1	2.6
phosphoglycerate mutase 1	Pgam1	5.3
phosphoglucomutase 1	Pgm1	2.6
phosphoglucomutase 2	Pgm2	2.2
enolase 1, alpha non-neuron	Eno1	6.0
pyruvate kinase, muscle	Pkm2	2.2
solute carrier family 2 (facilitated glucose transporter), or Glut3	Slc2a3	4.0
**Genes of aerobic glycolysis**		
lactate dehydrogenase A	Ldha	3.1
lactate dehydrogenase B	Ldhb	2.7
**Genes of citric acid cycle**		
pyruvate dehydrogenase kinase, isoenzyme 3	Pdk3	6.3
isocitrate dehydrogenase 1 (NADP+), soluble	Idh1	4.5
Succinate dehydrogenase complex, subunit B, iron sulfur (Ip	Sdhb	3.4
Succinate dehydrogenase complex, subunit B, iron sulfur (Ip	Sdhb	3.4
malate dehydrogenase 2, NAD (mitochondrial)	Mdh2	3.7
**Genes of glycogen synthesis**		
liver glycogen phosphorylase	Pygl	2.7
amylo-1,6-glucosidase, 4-alpha-glucanotransferase	Agl	5.9
NM_028132//phosphoglucomutase 2	Pgm2	2.2
UDP-glucose pyrophosphorylase 2	Ugp2	2.4
glycogen synthase 1, muscle	Gys1	6.1

### Gene expression levels of mitochondrial respiratory assembly and energy generating proteins in lal−/− MDSCs

**Figure 4:**
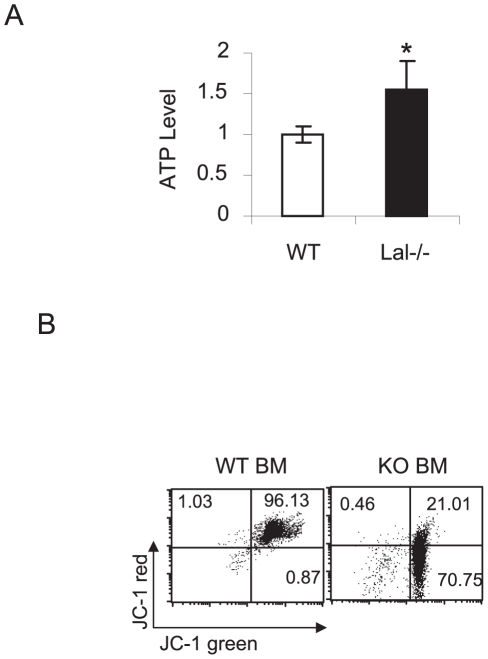
ATP production and mitochondrial potential analyses of lal−/− bone marrow MDSCs. **A**). ATP concentrations in Ly6G^+^ MDSCs from the bone marrow of *lal+/+* mice and *lal−/−* mice. Results are means of 4 independent experiments. n = 3, **, p<0.01, *, p<0.5; **B**). Mitochondrial membrane potential of CD11b^+^Ly6G^+^ MDSCs from the bone marrow of *lal+/+* mice and *lal−/−* mice was analyzed by JC-1 staining. Positive staining of JC-1 red represents healthy mitochondria. Transition from negative staining of JC-1 red to positive staining of JC-1 green represents impaired mitochondrial potential.

Mitochondria are the powerhouse for cells. In order to support robust cell proliferation, ATP synthesis is required. In mitochondrial oxidative phosphorylation, the synthesis of ATP is coupled to the flow of electrons from NADH or FADH2 to O_2_ by a proton gradient across the inner mitochondrial membrane and a series of electron carriers, such as NADH dehydrogenases and the cytochromes [23]. The synthesis of ATP is carried out by mitochondrial ATPase in the inner mitochondrial membrane. In *lal−/−* bone marrow MDSCs, the ATP concentration was significantly increased compared with wild type mice (Figure 4A). As demonstrated in Table 12, 13, expression of multiple NADH dehydrogenase subunits, cytochrome subunits, and mitochondrial ATPase were all up-regulated in *lal−/−* bone marrow MDSCs. Of the 85 mitochondrial respiratory chain subunits, 13 are synthesized within the organelle by the mitochondrial ribosome [24]. Gene expression of multiple mitochondrial ribosomal protein subunits were also up-regulated in *lal−/−* bone marrow MDSCs (Table S3), indicating the increased activity of protein synthesis within the mitochondria to meet the demand of ATP production. Despite increased mitochondrial activities and ATP synthesis, the membrane potential was reduced (Figure 4B), an indication of the functional impairment of mitochondria in *lal−/−* bone marrow MDSCs.

**Table 12:** Up and down-regulation of mitochondrial NADH dehydrogenases in *lal−/−* bone marrow MDSCs.

Genes	Symbol	Fold
NADH dehydrogenase (ubiquinone) Fe-S protein 5	Ndufs5	38.5
NAD(P)H dehydrogenase, quinone 2	Nqo2	6.3
NADH dehydrogenase Fe-S protein 5 pseudogene	BC002163	4.9
NADH dehydrogenase (ubiquinone) 1 beta subcomplex, 7	Ndufb7	4.4
NADH dehydrogenase (ubiquinone) flavoprotein 3	Ndufv3	4.3
NADH dehydrogenase (ubiquinone) 1 alpha subcomplex 11	Ndufa11	3.6
NADH dehydrogenase (ubiquinone) 1 beta subcomplex 8	Ndufb8	3.6
NADH dehydrogenase (ubiquinone) Fe-S protein 4	Ndufs4	3.2
NADH dehydrogenase (ubiquinone) 1 beta subcomplex, 6	Ndufb6	3.2
NADH dehydrogenase (ubiquinone) 1 alpha subcomplex 11	Ndufa11	3.2
NADH dehydrogenase (ubiquinone) 1 alpha subcomplex, 7	Ndufa7	3.1
NADH dehydrogenase (ubiquinone) 1 alpha subcomplex, 2	Ndufa2	3.0
NADH dehydrogenase (ubiquinone) 1 beta subcomplex 3	Ndufb3	2.9
NADH dehydrogenase (ubiquinone) 1 beta subcomplex 4	Ndufb4	2.9
NADH dehydrogenase (ubiquinone) 1 alpha subcomplex, 3	Ndufa3	2.8
NADH dehydrogenase (ubiquinone) 1 alpha subcomplex, 8	Ndufa8	2.7
NADH dehydrogenase (ubiquinone) 1 alpha subcomplex, 1	Ndufa1	2.5
NADH dehydrogenase (ubiquinone) Fe-S protein 6	Ndufs6	2.4
NADH dehydrogenase (ubiquinone) flavoprotein 1	Ndufv1	2.4
NADH dehydrogenase (ubiquinone) 1 beta subcomplex, 2	Ndufb2	2.4
NADH dehydrogenase (ubiquinone) 1 alpha subcomplex, 13	Ndufa13	2.3
NADH dehydrogenase (ubiquinone) 1 beta subcomplex, 9	Ndufb9	2.2
NADH dehydrogenase (ubiquinone) Fe-S protein 2//1 H3	Ndufs2	2.2
NAD(P)H dehydrogenase, quinone 1	Nqo1	2.2
NADH dehydrogenase (ubiquinone) 1 beta subcomplex, 10	Ndufb10	2.2
NADH dehydrogenase (ubiquinone) flavoprotein 2	Ndufv2	2.1
NADH dehydrogenase (ubiquinone) 1 alpha subcomplex, 12	Ndufa12	2.1
NADH dehydrogenase (ubiquinone) 1 beta subcomplex, 11	Ndufb11	2.0
NADH dehydrogenase (ubiquinone) 1 alpha subcomplex, asse	Ndufaf4	−2.6
NADH dehydrogenase subunit 1	ND1	−2.7
NADH dehydrogenase subunit 4L	ND4L	−4.0

**Table 13:** Up-regulation of cytochrome and ATP synthesis subunits in *lal−/−* bone marrow MDSCs.

Genes of cytochrome subunits	Symbol	Fold
cytochrome b-245, alpha polypeptide	Cyba	8.6
cytochrome b5 reductase 4	Cyb5r4	5.1
cytochrome c oxidase subunit VIb polypeptide 2	Cox6b2	3.4
cytochrome b5 reductase 4	Cyb5r4	3.4
ubiquinol-cytochrome c reductase (6.4 kD) subunit	Uqcr	3.3
ubiquinol-cytochrome c reductase core protein 1	Uqcrc1	3.4
cytochrome P450, family 51	Cyp51	3.1
cytochrome P450, family 4, subfamily f, polypeptide 18	Cyp4f18	3.1
cytochrome b-245, beta polypeptide	Cybb	2.9
cytochrome c-1	Cyc1	2.5
ubiquinol-cytochrome c reductase, complex III subunit VII	Uqcrq	2.5
cytochrome c oxidase, subunit VIIa 2	Cox7a2	2.4
P450 (cytochrome) oxidoreductase	Por	2.4
cytochrome c oxidase subunit IV isoform 1	Cox4i1	2.1

Genes of ATP synthase	Symbol	Fold
ATP synthase, H+ transporting, mitochondrial F1 complex, e	Atp5e	3.3
ATP synthase, H+ transporting, mitochondrial F0 complex, s	Atp5h	2.9
ATP synthase, H+ transporting, mitochondrial F1F0 complex,	Atp5k	2.5
ATP synthase, H+ transporting, mitochondrial F0 complex,	Atp5f1	2.1
ATP synthase, H+ transporting, mitochondrial F1 complex, d	Atp5d	2.1

## Discussion

LAL plays an important role in controlling inflammation. Genetic ablation of LAL function leads to systemic expansion of MDSCs. The aggressive behavior of MDSCs in *lal−/−* mice relies on their intrinsic malfunction. The gene profile analysis reveals the underneath mechanisms by which LAL regulates MDSCs development and homeostasis. It is surprising that MDSCs in *lal−/−* animal model share many characteristics used by cancer cells.

### Activation of G-protein superfamily

LAL deficiency leads to vast changes of the G-protein signaling pathways (both large and small GTPases) in *lal−/−* bone marrow MDSCs (Table 1, 2, 3, 4, 5). Especially, multiple components of small GTPases are up-regulated. Since G-protein signaling pathways are well known for their diverse functions in cells (such as glucose metabolism, ion channels, transcription, motility, and secretion) [25], [26], ups and downs of these genes exert their profound pathological influences on MDSCs development and homeostasis in *lal−/−* mice. Among changed small GTPases, 1) Ras GTPases activate the Raf/Mek/Erk pathway, which mediates cell growth and cell-cycle entry by phosphorylation of transcription factors, MNK (MAPK-interacting serine/threonine kinase) family of kinases, and the PI3K/AKT pathway to control cell survival, growth and metabolism. Multiple Ras GTPases downstream effectors were up-regulated (Table 3, 4, 5). For example, Erk (Mapk3) was up to 7.3 folds, and p38 (Mapk13) was up to 5.1 folds. We reported previously that the phosphorylation levels of both Erk and p38 are substantially increased in *lal−/−* bone marrow MDSCs [4]; 2) Rho GTPases organize actin cytoskeleton, cell adhesion and cell motility. They promote cell-cycle progression through G1 by regulating cyclin D1 and cyclin-dependent inhibitors p21 and p27 [19]; 3) Rab GTPases regulate receptor internalization, vesicle formation and trafficking to various cellular sites, including the nucleus, lysosome and plasma membrane. Through regulation of endocytic trafficking, they integrate multiple signaling pathways that are involved in cellular proliferation, apoptosis and migration. Gene expression of multiple Rab GTPase-regulated membrane vesicle traffic effectors that mediate vesicle intracellular transport, including actin-dependent motors (myosins) or microtubule-dependent motors (kinesins or dyneins) (Table S4), and tethering factors (exocyst, trafficking protein particle complex or TRAPPC, vacuole protein sorting or VPS) (Table S5) [27] were significantly altered in *lal−/−* bone marrow MDSCs. Farnesylation on G-proteins is a critical step for membrane binding and biological function. Farnesyltransferase expression that farnesylates Ras on the CAAX motif was increase 2 folds in *lal−/−* bone marrow MDSCs (Table 3, 4, 5).

### Cell cycle and Histone cluster genes

The second group of disregulation in *lal−/−* bone marrow MDSCs belongs to cell cycle genes (Table 6). These cell cycle related genes control proper progression of the cell cycle by checkpoints that sense possible defects during DNA synthesis and chromosome segregation [21]. It has been demonstrated that CDK1 (6.6 fold increase) alone can drive the cell cycle in most mammalian cells [21]. Up-regulation of Cdk2, Cdk5, Cdk9 suggests that *lal−/−* bone marrow MDSCs acquire specific needs for the activities of these Cdks during development and homeostasis. Importantly, all Cdk regulatory cyclins (A, B, D, E-type) that are required for phase entering of the cell cycle were up-regulated, suggesting constitutive mitogenic signaling and defective responses to anti-mitogenic signals under the pressure of Ras GTPases overactivation that contribute to unscheduled proliferation during LAL deficiency. Up-regulation of enzymes/protein factors that are involved in ubiquitination and proteasome (Table 7 & 8) further suggests the higher cell cycle activity in *lal−/−* bone marrow MDSCs. Ubiquitin and ubiquitin-like molecules direct proteins to proteolysis within proteasome for recycling, which is required in a wide variety of cellular processes, including cell cycle and division, DNA transcription and repair, biogenesis of organelles, modulation of cell surface receptors, ion channels and the secretory pathway, response to stress and extracellular modulators, ribosome biogenesis [21], [28].

Another evidence of enhanced cell proliferation in *lal−/−* bone marrow MDSCs is up-regulation of multiple members of histone cluster genes (Table 9). Changes of these histone variants and associate factors set up an epigenetic microenvironment that preferentially favor certain gene expression and cellular events to promote MDSCs expansion in *lal−/−* mice. In addition to control gene expression, histone-variants exchange activities contribute to formation of centromeric and telomeric chromatin during cell cycles. A centromere is a region of DNA on chromosome where two identical sister chromatids come closest in contact. It is involved in cell division as the point of mitotic spindle attachment. During mitotic division, kinetochore is formed on top of the centromeres. The kinetochores are the sites where the spindle fibers attach. Kinetochores and the spindle apparatus are responsible for the movement of the two sister chromatids to opposite poles of dividing cell nucleus during anaphase. Up-regulation of centromeric and telomeric histones along with other M phase chromosome structural proteins demonstrated the increased activity of cell division in *lal−/−* bone marrow MDSCs (Table 10). This was further supported by the cell cycle analysis, in which *lal−/−* bone marrow MDSCs in G1/M phases were around 4 times more compared with wild type MDSCs (Figure 3).

### Metabolism and bioenergetics

Another similarity between *lal−/−* bone marrow MDSCs and cancer cells is reprogramming of energy metabolism. First, cells rely on mitochondrial oxidative phosphorylation to provide energy (ATP production) for cellular activities in normal conditions. Cancer cells are characterized by increased glycolysis and reduced mitochondrial respiratory function [24]. The electron flow through the respiratory chain is substantially lower in malignant cells leading to oxidative stress and increased ROS production. Similar to cancer cells, multiple enzymes and proteins in glycolysis and citric acid cycles were up-regulated in *lal−/−* bone marrow MDSCs (Table 11). Interestingly, the mitochondrial function in *lal−/−* bone marrow MDSCs was impaired (Figure 4B) despite increased expression of respiratory chain proteins (including NADH dehydrogenases, cytochrome proteins, ATPases and mitochondrial ribosomal proteins) (Table 12, 13) and increased ATP production (Figure 4 A) compared with those in wild type counterparts. Second, pyruvate (the end product of glycolysis) is imported into mitochondria and enters the citric acid cycle in normal cells. Cancer cells use an altered metabolic pattern by taking up much more glucose and mainly process it through aerobic glycolysis, producing large quantities of secreted lactate by lactate dehydrogenase (LDH) with a lower use of oxidative phosphorylation [23], [29]. This metabolic switch by aerobic glycolysis is advantageous to cancers cells to allow them better surviving, producing intermediates for cell growth and division. Similar to cancer cells, *lal−/−* bone marrow MDSCs showed increased gene expression of both lactate dehydrogenase A and B (Table 11), which keep pyruvate away from the mitochondria. This observation indicates that LAL-controlled neutral lipid metabolism plays a critical role in preventing metabolic switch from oxidative phosphorylation to aerobic glycolysis in myeloid lineage cells.

In addition to generate energy for the cell (ATP) and produce substrates to synthesize amino acids, nucleosides, and fatty acids, normal cells use both glucose and glutamine as substrates to regulate the redox potential to minimize the effects of reactive oxygen species (ROS) that damage membranes, proteins and cause mutations in a cell. Similar to cancer cells, the concentration of ROS was significantly increased in *lal−/−* bone marrow MDSCs (Figure 2B), which was accompanied by up-regulation of nitric oxide/ROS production genes (Figure 2A), glutathione peroxidase/glutathione reductase genes, and glucose 6-phosphate dehydrogenase gene (Table S2). High levels of ROS allow for the stimulation of cell proliferation, induction of genetic instability, and evasion from senescence [23].

### The mTOR pathway

Ingenuity Pathway Analysis revealed alteration in PI3K/thymoma viral proto-oncogene (AKT)/mammalian target of rapamycin (mTOR) signaling pathway [30], [31], [32] in *lal−/−* bone marrow MDSCs (Figure 1B). Activation of the mTOR pathway has been confirmed by highly phosphorylated S6 and 4E-BP, two authentic mTOR downstream effectors (Figure 1C). mTOR serves as a signal integrator for nutrients, growth factors, energy and stress [20]. Activation of this pathway suppresses apoptosis, promotes an influx of glucose and amino acids into the cells, stimulates ATP production [17], as well as contributes to cell growth, cell cycle entry, cell survival, and cell motility during tumorigenesis [33]. Increasing evidence suggests that membrane trafficking causes mTORC1 to shuttle to lysosomes and regulate mTORC1signalling, enabling it to respond to growth factors [20], [34]. The lysosomal surface hosts a molecular machinery for mTORC1 activation that includes the Rag GTPases, the trimeric regulator complex, and possibly GTPase activating proteins (GAPs) and guanine nucleotide exchange factors (GEFs) for the Rag GTPases [20]. Since LAL is a lysosome-associated enzyme, it is conceivable that lack of the LAL activity may change lipid composition and dynamics on the lysosomal membrane that influence endomembrane trafficking and stimulate the mTOR1 activity, which in tune coordinates the cellular metabolism and growth to increase abnormal proliferation of *lal−/−* bone marrow MDSCs.

It is important to keep in mind that development and expansion of MDSCs are a complex process. In addition to changes of gene expression, posttranscriptional modification of intracellular signaling pathways also contributes to the *lal−/−* MDSCs autonomous defect. For example, although up-regulation of Stats family members was not detected by Affymetrix GeneChip microarray analysis, phosphorylation of Stat3 at Y705 has been detected in expanded *lal−/−* MDSCs [4]. Activation of Stat3 directly leads to MDSCs expansion in vivo [35], [36]. Phosphorylation of Erk and p38 in the Ras signaling pathway has also been detected in expanded *lal−/−* MDSCs.

In summary, studies outlined here demonstrate that the loss of the LAL function leads to myeloproliferative neoplasm. Affymetrix GeneChip microarray analysis provides a detailed map of intrinsic defects existing in *lal−/−* bone marrow MDSCs. The interrelationships between these biological processes during *lal−/−* MDSCs development and homeostasis remain to be elucidated in the future. The study provides new avenues and interventions clinically to eliminate MDSCs expansion in disease setting.

## Supporting Information

Table S1 **Changes of histone related genes in MDSCs from the bone marrow of** ***lal−/−*** **mice.**
(DOC)

Table S2 **Up-regulation of metabolic enzyme genes in the pentose pathway in MDSCs from the bone marrow of** ***lal−/−*** **mice.**
(DOC)

Table S3 **Up-regulation of mitochondrial ribosomal protein subunits in** ***lal−/−*** **bone marrow MDSCs.**
(DOC)

Table S4 **Changes of vesicle traffic motor genes in MDSCs from the bone marrow of** ***lal−/−*** **mice.**
(DOC)

Table S5 **Changes of vesicle traffic tethering factor and membrane fusion factor genes in MDSCs from the bone marrow of** ***lal−/−*** **mice.**
(DOC)
