# Supplementary material for: Gene Profile of Myeloid-Derived Suppressive Cells from the Bone Marrow of Lysosomal Acid Lipase Knock-Out Mice
Source: PLoS One. 2012 Feb 27;7(2):e30701. doi: 10.1371/journal.pone.0030701 (PMC3288004; doi:10.1371/journal.pone.0030701)
Supplement: Table S1 — Changes of histone related genes in MDSCs from the bone marrow of lal−/− mice. (DOC) [file pone.0030701.s001.doc]

Table S1, Changes of histone related genes in MDSCs from the bone marrow of *lal-/-* mice.

| **Genes** | **Symbol** | **Fold** |
| --- | --- | --- |
| centrosomal protein 55 | Cep55 | 4.9 |
| nucleolar and spindle associated protein 1 | Nusap1 | 3.8 |
| structural maintenance of chromosomes 2 | Smc2 | 3.6 |
| SET domain containing (lysine methyltransferase) 8 | Setd8 | 3.5 |
| spindle assembly 6 homolog (C. elegans) | Sass6 | 3.5 |
| H2-K region expressed gene 2 | H2-Ke2 | 3.3 |
| methyltransferase like 9 | Mettl9 | 3.2 |
| chromatin modifying protein 4B | Chmp4b | 2.6 |
| protection of telomeres 1A | Pot1a | 2.5 |
| chromatin modifying protein 2A | Chmp2a | 2.4 |
| histone deacetylase 1 | Hdac1 | 2.4 |
| inner centromere protein | Incenp | 2.3 |
| X B|X 28.85 cM | Cetn2 | 2.3 |
| chromatin modifying protein 5 | Chmp5 | 2.3 |
| regulator of chromosome condensation (RCC1) and BTB (POZ) | Rcbtb2 | 2.2 |
| methyltransferase like 10 | Mettl10 | 2.2 |
| chromatin accessibility complex 1 | Chrac1 | 2.2 |
| centrosome and spindle pole associated protein 1 | Cspp1 | 2.1 |
| chromatin modifying protein 1B | Chmp1b | 2.1 |
| chromodomain helicase DNA binding protein 7 | Chd7 | 2.0 |
|  |  |  |
| regulator of chromosome condensation (RCC1) and BTB (POZ) | Rcbtb1 | -2.4 |
| histone deacetylase 9 | Hdac9 | -2.2 |

| ADP-ribosylation factor-like 2 binding protein | Arl2bp | 5.0 |
| --- | --- | --- |
| ADP-ribosylation factor 1 | Arf1 | 3.8 |
| ADP-ribosylation factor-like 8A | Arl8a | 3.4 |
| ADP-ribosylation factor-like 15 | Arl15 | 3.3 |
| ADP-ribosylation factor-like 11 | Arl11 | 2.8 |
| ADP-ribosylation factor-like 6 interacting protein 6 | Arl6ip6 | 2.6 |
| ADP-ribosylation factor guanine nucleotide-exchange f | Arfgef1 | 2.5 |
| ADP-ribosylation factor interacting protein 1 | Arfip1 | 2.4 |
| ADP-ribosylation factor-like 5B | Arl5b | 2.2 |
| ADP-ribosylation factor GTPase activating protein 2 | Arfgap2 | 2.1 |
| ADP-ribosylation factor related protein 1 | Arfrp1 | 2.0 |
| ADP-ribosylation factor-like 9 | Arl9 | -2.4 |
