# Supplementary material for: Gene Profile of Myeloid-Derived Suppressive Cells from the Bone Marrow of Lysosomal Acid Lipase Knock-Out Mice
Source: PLoS One. 2012 Feb 27;7(2):e30701. doi: 10.1371/journal.pone.0030701 (PMC3288004; doi:10.1371/journal.pone.0030701)
Supplement: Table S2 — Up-regulation of metabolic enzyme genes in the pentose pathway in MDSCs from the bone marrow of lal−/− mice. (DOC) [file pone.0030701.s002.doc]

Table S2. Up-regulation of metabolic enzyme genes in the pentose pathway in MDSCs from the bone marrow of *lal-/-* mice.

| **Genes of pentose phosphate pathway** | **Symbol** | **Fold** |
| --- | --- | --- |
| glucose-6-phosphate dehydrogenase 2 | G6pd2 | 5.0 |
| glucose-6-phosphate dehydrogenase X-linked | G6pdx | 5.7 |
| phosphogluconate dehydrogenase | Pgd | 4.6 |
| transketolase | Tkt | 7.3 |
| transaldolase 1 | Taldo1 | 2.6 |
| ribulose-5-phosphate-3-epimerase | Rpe | 3.7 |
| glutathione reductase | Gsr | 20.1 |
| glutathione peroxidase 4 | Gpx4 | 2.9 |
