# Supplementary material for: Gene Profile of Myeloid-Derived Suppressive Cells from the Bone Marrow of Lysosomal Acid Lipase Knock-Out Mice
Source: PLoS One. 2012 Feb 27;7(2):e30701. doi: 10.1371/journal.pone.0030701 (PMC3288004; doi:10.1371/journal.pone.0030701)
Supplement: Table S3 — Up-regulation of mitochondrial ribosomal protein subunits in lal−/− bone marrow MDSCs. (DOC) [file pone.0030701.s003.doc]

Table S3. Up-regulation of mitochondrial ribosomal protein subunits in *lal-/-* bone marrow MDSCs.

| **Genes** | **Symbol** | **Fold** |
| --- | --- | --- |
| mitochondrial ribosomal protein L45 | Mrpl45 | 4.0 |
| mitochondrial ribosomal protein S18A | Mrps18a | 3.8 |
| mitochondrial ribosomal protein S15 | Mrps15 | 3.7 |
| mitochondrial ribosomal protein S35 | Mrps35 | 3.6 |
| mitochondrial ribosomal protein L18 | Mrpl18 | 3.6 |
| mitochondrial ribosomal protein L48 | Mrpl48 | 3.1 |
| mitochondrial ribosomal protein S21 | Mrps21 | 3.1 |
| mitochondrial ribosomal protein L19 | Mrpl19 | 2.7 |
| mitochondrial ribosomal protein L20 | Mrpl20 | 2.7 |
| mitochondrial ribosomal protein 63 | Mrp63 | 2.6 |
| mitochondrial ribosomal protein L41 | Mrpl41 | 2.3 |
| mitochondrial ribosomal protein S23 | Mrps23 | 2.2 |
| mitochondrial ribosomal protein L13 | Mrpl13 | 2.2 |
| mitochondrial ribosomal protein S18C | Mrps18c | 2.2 |
| mitochondrial ribosomal protein L27 | Mrpl27 | 2.1 |
| mitochondrial ribosomal protein L44 | Mrpl44 | 2.1 |
| mitochondrial ribosomal protein L14 | Mrpl14 | 2.0 |
| mitochondrial ribosomal protein L40 | Mrpl40 | 2.0 |
