# Supplementary material for: Gene Profile of Myeloid-Derived Suppressive Cells from the Bone Marrow of Lysosomal Acid Lipase Knock-Out Mice
Source: PLoS One. 2012 Feb 27;7(2):e30701. doi: 10.1371/journal.pone.0030701 (PMC3288004; doi:10.1371/journal.pone.0030701)
Supplement: Table S4 — Changes of vesicle traffic motor genes in MDSCs from the bone marrow of lal−/− mice. (DOC) [file pone.0030701.s004.doc]

Table S4. Changes of vesicle traffic motor genes in MDSCs from the bone marrow of *lal-/-* mice.

| **Genes** | **Symbol** | **Fold** |
| --- | --- | --- |
| myosin, light polypeptide 2, regulatory, cardiac, slow | Myl2 | 4.0 |
| myosin VA | Myo5a | 2.3 |
| myosin, heavy polypeptide 9, non-muscle | Myh9 | 2.3 |
| myosin IF | Myo1f | 2.3 |
| myosin, light polypeptide 6, alkali, smooth muscle and non- | Myl6 | 2.2 |
| myosin IG | Myo1g | 2.1 |
| similar to myosin regulatory light chain-like | LOC100048581 | 2.1 |
| myosin X | Myo10 | -2.0 |
| Myl4 | Myl4 | -3.4 |
| Myo9a | Myo9a | -4.0 |
| myosin IE | Myo1e | -6.7 |
| kinesin family member 15 | Kif15 | 6.2 |
| kinesin family member 11 | Kif11 | 5.9 |
| kinesin family member 4 | Kif4 | 5.0 |
| kinesin family member 22 | Kif22 | 4.0 |
| kinesin family member 2C | Kif2c | 3.8 |
| trafficking protein, kinesin binding 2 | Trak2 | 3.4 |
| kinesin family member 14 | Kif14 | 2.6 |
| kinesin family member 18A | Kif18a | 2.5 |
| kinesin family member 3B | Kif3b | 2.3 |
| kinesin family member 20A | Kif20a | 2.1 |
| dynein cytoplasmic 1 intermediate chain 2 | Dync1i2 | 3.0 |
| dynein light chain Tctex-type 3 | Dynlt3 | 2.6 |
| dynein cytoplasmic 1 light intermediate chain 1 | Dync1li1 | 2.4 |
| dynein, axonemal, heavy chain 8 | Dnahc8 | 2.2 |
| dynein light chain Tctex-type 1 | Dynlt1 | 2.2 |
| dynein cytoplasmic 2 light intermediate chain 1 | Dync2li1 | 2.1 |
