# Supplementary material for: Gene Profile of Myeloid-Derived Suppressive Cells from the Bone Marrow of Lysosomal Acid Lipase Knock-Out Mice
Source: PLoS One. 2012 Feb 27;7(2):e30701. doi: 10.1371/journal.pone.0030701 (PMC3288004; doi:10.1371/journal.pone.0030701)
Supplement: Table S5 — Changes of vesicle traffic tethering factor and membrane fusion factor genes in MDSCs from the bone marrow of lal−/− mice. (DOC) [file pone.0030701.s005.doc]

Table S5. Changes of vesicle traffic tethering factor and membrane fusion factor genes in MDSCs from the bone marrow of *lal-/-* mice.

| **Gene** | **Symbol** | **Fold** |
| --- | --- | --- |
| ***Tethering factors*** |  |  |
| exocyst complex component 1 | Exoc1 | 3.8 |
| exocyst complex component 3 | Exoc3 | 3.2 |
| exocyst complex component 6 | Exoc6 | 3.2 |
| exocyst complex component 2 | Exoc2 | 3.0 |
| exocyst complex component 5 | Exoc5 | 2.1 |
| trafficking protein particle complex 2 | Trappc2 | 3.6 |
| trafficking protein particle complex 1 | Trappc1 | 2.8 |
| trafficking protein particle complex 10 | Trappc10 | 2.1 |
| trafficking protein particle complex 4 | Trappc4 | 4.9 |
| trafficking protein particle complex 3 | Trappc3 | 4.1 |
| trafficking protein particle complex 2-like | Trappc2l | 4.0 |
| vacuolar protein sorting 26 homolog B (yeast) | Vps26b | 4.6 |
| vacuolar protein sorting 29 (S. pombe) | Vps29 | 3.9 |
| vacuolar protein sorting 25 (yeast) | Vps25 | 3.3 |
| Vps20-associated 1 homolog (S. cerevisiae) | Vta1 | 3.2 |
| vacuolar protein sorting 24 (yeast) | Vps24 | 3.2 |
| vacuolar protein sorting 28 (yeast) | Vps28 | 3.1 |
| vacuolar protein sorting 36 (yeast) | Vps36 | 3.0 |
| vacuolar protein sorting 13 D (yeast) | Vps13d | 2.8 |
| vacuolar protein sorting 4a (yeast) | Vps4a | 2.8 |
| vacuolar protein sorting 45 (yeast) | Vps45 | 2.7 |
| vacuolar protein sorting 35 | Vps35 | 2.7 |
| vacuolar protein sorting 37C (yeast) | Vps37c | 2.3 |
| vacuolar protein sorting 26 homolog A (yeast) | Vps26a | 2.1 |
| vacuolar protein sorting 54 (yeast) | Vps54 | 2.0 |
| vacuolar protein sorting 4b (yeast) | Vps4b | 2.0 |
| ***Membrane fusion*** |  |  |
| vesicle transport through interaction with t-SNAREs homolog | Vti1a | 5.4 |
| vesicle transport through interaction with t-SNAREs 1B homolog | Vti1b | 2.3 |
